# Alcohol intake and risk of colorectal cancer: Results from the UK Dietary Cohort Consortium

**DOI:** 10.1038/sj.bjc.6605802

**Published:** 2010-07-20

**Authors:** J Y Park, C C Dahm, R H Keogh, P N Mitrou, B J Cairns, D C Greenwood, E A Spencer, I S Fentiman, M J Shipley, E J Brunner, J E Cade, V J Burley, G D Mishra, D Kuh, A M Stephen, I R White, R N Luben, A A Mulligan, K-T Khaw, S A Rodwell

**Affiliations:** 1 Department of Public Health and Primary Care, University of Cambridge, Cambridge CB1 8RN, UK; 2 Department of Public Health and Primary Care, Medical Research Council Centre for Nutritional Epidemiology in Cancer Prevention and Survival, University of Cambridge, Cambridge CB1 8RN, UK; 3 Medical Research Council Biostatistics Unit, Institute of Public Health, University of Cambridge, Cambridge CB2 0SR, UK; 4 Cancer Epidemiology Unit, University of Oxford, Oxford OX3 7LF, UK; 5 Centre for Epidemiology and Biostatistics, Faculty of Medicine and Health, University of Leeds, Leeds LS2 9JT, UK; 6 Academic Oncology Unit, Guy's Hospital, London, SE1 9RT, UK; 7 Department of Epidemiology and Public Health, University College London, London WC1E 6BT, UK; 8 Department of Epidemiology and Public Health, Medical Research Council Unit for Lifelong Health and Ageing, University College London, 33 Bedford Place, London WC1B 5JU, UK; 9 Medical Research Council Human Nutrition Research, Elsie Widdowson Laboratory, Cambridge CB1 9NL, UK

**Keywords:** colorectal cancer, alcohol intake, prospective cohort study, food diary, food frequency questionnaire

## Abstract

**Background:**

Epidemiological studies have suggested that excessive alcohol intake increases colorectal cancer (CRC) risk. However, findings regarding tumour subsites and sex differences have been inconsistent.

**Methods:**

We investigated the prospective associations between alcohol intake on overall and site- and sex-specific CRC risk. Analyses were conducted on 579 CRC cases and 1996 matched controls nested within the UK Dietary Cohort Consortium using standardised data obtained from food diaries as a main nutritional method and repeated using data from food frequency questionnaire (FFQ).

**Results:**

Compared with individuals in the lightest category of drinkers (>0–<5 g per day), the multivariable odds ratios of CRC were 1.16 (95% confidence interval (95% CI): 0.88, 1.53) for non-drinkers, 0.91 (95% CI: 0.67, 1.24) for drinkers with 5–<15 g per day, 0.90 (95% CI: 0.65, 1.25) for drinkers with 15–<30 g per day, 1.02 (95% CI: 0.66, 1.58) for drinkers with 30–<45 g per day and 1.19 (95% CI: 0.75, 1.91) for drinkers with ⩾45 g per day. No clear associations were observed between site-specific CRC risk and alcohol intake in either sex. Analyses using FFQ showed similar results.

**Conclusion:**

We found no significantly increased risk of CRC up to 30 g per day of alcohol intake within the UK Dietary Cohort Consortium.

The descriptive epidemiology of colorectal cancer (CRC) shows significant geographical variation in incidence rates worldwide and provides strong circumstantial evidence that lifestyle has an important role in colorectal carcinogenesis (Stewart and Kleihues, 2003). Alcohol drinking is one such important lifestyle factor (Ferrari *et al*, 2007): the IARC (International Agency for Research on Cancer) recently added CRC to the list of alcohol-related malignancies (Baan *et al*, 2007), and the 2007 World Cancer Research Fund/American Institute for Cancer Research Expert Report (WCRF/AICR Report) concluded that intake of ethanol from alcoholic drinks of >30 g per day is a convincing cause of CRC in men and a probable cause in women (WCRF/AICR, 2007). In the United Kingdom, 30 g of alcohol is equivalent to 3–4 units, 1 unit being ∼8 g of alcohol (The National Health Service, 2010). Associations between alcohol intake and CRC risk according to anatomical subsites of the colorectum remain unclear (Chen *et al*, 2005; Akhter *et al*, 2007; Ferrari *et al*, 2007; Bongaerts *et al*, 2008; Lim and Park, 2008), although it is believed that colon and rectal cancers have different aetiologies (Li and Lai, 2009), and that within the colon, proximal and distal sites have biologically distinct functions (Bufill, 1990; Lindblom, 2001). Evidence has mostly been available for men with high alcohol intake (Allen *et al*, 2009), and risks of CRC with alcohol intake for men and women have not been consistent.

Many epidemiological studies which investigated an effect of alcohol on health have relied on self-reports of alcohol intakes. Owing to its simplicity in use and convenience in administration, food frequency questionnaires (FFQs) have been mostly used in alcohol intake assessment (Feunekes *et al*, 1999). However, as a nutritional instrument, FFQs may have both large random and systematic measurement errors (Bingham *et al*, 2003; Prentice, 2003; Schatzkin *et al*, 2003), and for a number of nutrients, food diaries have been shown to provide measurements that are more strongly associated with biomarker data (Bingham *et al*, 1997, 2008; Day *et al*, 2001). Furthermore, it has been suggested that food diaries can capture a more complicated individual dietary intake more accurately (Bingham *et al*, 2003). However, less is known about whether food diaries provide a superior measure of food intake for infrequently or episodically consumed items, such as alcoholic drinks, compared with the FFQs. Therefore, it is important to compare the effects of alcohol intake on CRC risk using food diaries and FFQs.

In the United Kingdom, government recommendations on alcohol intake are for men to consume no more than 3–4 units per day (<32 g per day) and for women to consume no more than 2–3 units per day (<24 g per day) (The National Health Service, 2010); however, the average annual alcohol intake in the United Kingdom now exceeds the European Union average (Department of Health, 2009) and CRC is the second major cause of cancer death in the country (Westlake and Cooper, 2008). Worldwide, more than one million incident cases were recorded in 2002 (WCRF/AICR, 2007). Hence, even a moderate association between alcohol intake and CRC risk may have important public health implications.

The aim of this study was to examine the relationship between alcohol intake and overall and site-specific CRC risks, including differences in sex-specific risks, using a case–control study nested within the UK Dietary Cohort Consortium, from which nutritional data were ascertained by food diaries and FFQs at baseline.

## Materials and methods

### Study population

The UK Dietary Cohort Consortium comprises seven established UK cohorts (namely EPIC-Norfolk, EPIC-Oxford, Guernsey Study, Oxford Vegetarian Study, MRC National Survey of Health and Development (NSHD), the UK Women's Cohort Study (UKWCS) and Whitehall II; Table 1) with a total cohort size of 153 000 individuals. The methods of recruitment, study design and ethical approval have been described for each of these cohorts in detail elsewhere (Appleby *et al*, 1999; Day *et al*, 1999; Davey *et al*, 2003; Cade *et al*, 2004; Allen *et al*, 2005; Marmot and Brunner, 2005; Wadsworth *et al*, 2006).

### Case ascertainment

Case patients were individuals who were free of cancer (except non-melanoma skin cancer) at the date of food diary commencement and who developed CRC at least 12 months after the date of diary commencement and before the end of the study period, defined for each study centre by the latest date of complete follow-up for both cancer incidence and vital status.

The last dates of follow-up varied between cohorts, from 31 December 2003 to 1 January 2007. Individuals with self-reported or registry-reported prevalent cancer (except non-melanoma skin cancer) were omitted from the study. Incident CRC cases (International Statistical Classification of Diseases and Related Health Problems (ICD) 10th Revision, C18–20) were ascertained by record linkage with local cancer registries and the United Kingdom Office for National Statistics, which provided notification of all cancer registrations and deaths by cause for the cohort. For this study, CRC cases were classified according to anatomical subsites: colon cancers were defined as tumours in the caecum, appendix, ascending colon and hepatic flexure, transverse colon, splenic flexure (proximal, C18.0–18.5; ICD 10th Revision), and descending and sigmoid colon (distal, C18.6–C18.7), as well as tumours that were overlapping or unspecified (C18.8 and C18.9). Cancer of the rectum included tumours occurring at the rectosigmoid junction (C19) and rectum (C20). Overall CRC was defined as a combination of all colon and rectal cancer cases.

### Selection of matched controls

Cases were matched within their respective cohort to four controls each, with the exception of some cases from EPIC-Oxford, the Guernsey Study and the Oxford Vegetarian Study who were matched to two controls, and some from the UKWCS who were matched to five controls. Matched controls were selected at random from the appropriate stratum of the set of all cohort members who were free of CRC at the end of follow-up (due to death or censoring) and free of all cancer (except non-melanoma skin cancer) at the date of diary commencement. Matching criteria were sex, age at enrolment (±3 years) and month of diary completion (±3 months). Follow-up time for matched controls was also required to be at least as long as that for the case, with follow-up time defined as the time from the date of diary commencement to the date of CRC diagnosis for cases and the time from date of diary commencement until the end of follow-up for controls. A total of 579 CRC cases and 1996 matched controls were available for analysis.

### Diet and lifestyle assessment

Each cohort collected dietary information using 4-day (Guernsey, Oxford Vegetarian Study, UKWCS) (Appleby *et al*, 1999; Cade *et al*, 2004) or 7-day food diaries (EPIC-Norfolk, NSHD, EPIC-Oxford, Whitehall II) (Bingham *et al*, 2001; Brunner *et al*, 2001; Davey *et al*, 2003; Wadsworth *et al*, 2006) completed on consecutive days at recruitment to the study or during a subsequent monitoring phase. Participants were asked to record in detail all the foods and beverages they consumed, prompted by time slots such as ‘Mid-morning – between breakfast time and lunchtime’ and also by photographs of standard plates with three different portion sizes of representative foods to help participants estimate the amounts they consumed (Bingham *et al*, 2001). Information on age, sex, height, weight, smoking status, educational level, social class, physical activity and family history of CRC, were collected either by trained researchers or in questionnaires administered before the completion of the food diary. In four of the seven studies (namely EPIC-Norfolk, EPIC-Oxford, UKWCS and Whitehall II), FFQs were also administered before this data collection, and were available for analysis from most participants in these cohorts. The FFQs were based on that used in the US Nurses' Health Study, listed from 127 to 217 items, and have been validated for use in the United Kingdom (Bingham *et al*, 1997; Brunner *et al*, 2001; Cade *et al*, 2004).

The majority of data from the food diaries were coded to give nutrient intakes and food group information using data entry program Data Into Nutrients for Epidemiological Research (DINER) developed in the EPIC-Norfolk cohort (Welch *et al*, 2001). A total of 107 UKWCS food diaries were coded and processed using the Diet and Nutrition Tool for Evaluation (DANTE) program (Cade *et al*, 2006). We compared 100 food diaries coded under both systems and found good agreement between DANTE and DINER for most nutrients, although the geometric mean intake of alcohol from DINER was 7% higher (95% confidence interval (95% CI): 3–11%) than from DANTE.

### Alcohol intake assessment

For the food diaries completed by all centres, beer (stout, bitter, lager; keg, draught, bottled, canned; low alcohol, strong, home-made; number of pints, bottles, cans), cider (sweet, dry, vintage, low alcohol; number of pints, bottles, cans), spirits (what sort: e.g., whisky, gin, vodka, rum; at home or in a pub; single measures as in pub), wine, sherry, port (white, red; sweet, medium, dry; low alcohol; glasses) were assessed for alcohol intake.

The FFQs from EPIC-Norfolk, EPIC-Oxford, UKWCS and Whitehall II were designed to measure a participant's usual food intake during the previous year. In the four centres, FFQs asked participants to estimate how often they drink the following the beverages, ‘Beer, larger or cider (half pint)’, ‘Port, sherry, vermouth, liqueurs (glass)’ and ‘Spirits, e.g. gin, brandy, whisky, vodka (single)’. For each item on the list, participants were asked to indicate their usual intake, choosing from nine frequency categories, ranging from ‘never or less than once per month’ to ‘more than 6 times per day’.

### Statistical analysis

Conditional logistic regression models were used to estimate odds ratios (ORs) and 95% CIs for the CRC risk according to alcohol intake, with adjustment for potential confounding variables.

The participants were categorised into six groups according to their baseline alcohol intake, with the lightest category of drinkers (>0–<5 g per day) as a reference group: 0 (non-drinkers), >0–<5, 5–<15, 15–<30, 30–<45, ⩾45 g per day. An initial unadjusted model was first created to estimate ORs for CRC across categories of alcohol intake. As the matching of cases and controls by age was not exact, the conditional logistic regression models were adjusted for age in years to control for any residual confounding. Multivariable model 1 also adjusted for intakes of energy (kcal per day), folate (*μ*g per day), dietary fibre (g per day), red meat (g per day), and processed meat (g per day) in addition to height (m), weight (kg), smoking status (never, former, current) and social class (six categories). There were some missing data within studies, with ∼1% of individuals missing weight, height and smoking status, and ∼5% missing social class, all of which were recorded in all studies. The distribution of alcohol intake among individuals with and without these missing data was similar. For these variables, missing values were assumed to be missing at random and were imputed using multiple imputation. In all, 10 imputed data sets were created and multivariable models were fitted using the ‘ice’ (Royston, 2005) and ‘mim’ (Carlin, 2008) packages in STATA (Stata Corporation, College Station, TX, USA). Multivariable model 2 adjusted for physical activity (inactive, moderately inactive, moderately active and active) and educational level (none, GCSE (completed to age 15 years), A Level (completed to age 17 years) and degree level) in addition to the adjustments in multivariable model 1. Data on physical activity level were not available for NSHD and the Guernsey Study, and information on educational level was not available for the Oxford Vegetarian Study. The effects of adjustment for these variables were assessed by fitting multivariable models 1 and 2 using the subset of participants (458 cases and 1734 controls) with complete information on physical activity and educational level. Sex-specific and anatomical subsite-specific models were also fitted using multivariable models 1 (579 cases and 1996 controls) and 2 (458 cases and 1734 controls). Tumours that were overlapping or unspecified were not included in site-specific analyses of the proximal and the distal colon cancer (*n*=60).

To investigate whether different nutritional instruments might alter our results, we repeated the analyses using FFQ data. Dietary data obtained from FFQs were available for participants in EPIC-Norfolk, EPIC-Oxford, the UKWCS and Whitehall II (496 cases and 1809 controls). These analyses were restricted to those 2305 participants who completed both the FFQ and the food diary, and ORs were estimated using multivariable models 1 and 2.

Tests for trend were conducted by modelling alcohol intake as a continuous variable in a conditional logistic regression analysis. To assess the possibility of a non-linear association between alcohol intake and CRC risk, the multivariable models were fitted with the inclusion of a quadratic term for continuous alcohol intake. Simple associations between categorical covariates and alcohol intake were assessed using Pearson's *χ*^2^ tests for two independent proportions. For continuous variables, means across categories of alcohol intake were compared by *t*-tests, analysis of variance or a Kruskal–Wallis test (for red meat and processed meat intake only). All statistical tests were two sided, and all statistical analyses were performed using the statistical software package STATA (version 10).

## Results

A total of 579 incident CRC cases and 1996 matched controls were available for analysis from the 7 participating UK cohorts. Of these cancer cases, 380 were located in the colon and 199 in the rectum. There were no statistically significant differences in the means of alcohol intake between cases and controls in each cohort (Table 1).

Table 2 presents participant characteristics according to categories of alcohol intake. Among drinkers, 82% consumed <30 g per day alcohol. The average alcohol intake was ∼17 g per day (∼2 units per day) for men and 8 g per day (1 unit per day) for women. Men less frequently reported being non-drinkers and more frequently reported drinking ⩾30 g per day of alcohol than did women. Men who consumed ⩾30 g per day were significantly younger and had slightly higher BMI compared with those who consumed <30 g per day. These men with ⩾30 g per day of alcohol intake more frequently reported being former or current smokers, had higher energy intake, were physically less active and had attained a higher educational level, as well as being more likely to be non-manual workers. Similar patterns were seen among women, although women with ⩾30 g per day of alcohol intake had a lower mean BMI compared with non-drinkers, and there was no significant difference in physical activity levels across categories of alcohol intake.

Table 3 shows the ORs for CRC by categories of alcohol intake as assessed by the food diary from age-adjusted and multivariable models. Non-drinkers had a moderate, non-significant increased risk compared with those who drank >0–<5 g per day (<1 units per day) in the main models. As we were unable to differentiate individuals who did not drink in the time period during which their food diaries were recorded from never drinkers (former drinkers or life-long never drinkers), the category of non-drinkers might include temporary non-drinkers who are in fact drinkers. Therefore, we focused on analyses from individuals who reported non-zero alcohol intake.

In general, alcohol intake was not significantly associated with the risk of CRC (Table 3). Compared with individuals in the lowest category of alcohol intake among drinkers (>0–<5 g per day), individuals in the highest category of intake who consumed ⩾45 g per day (∼6 units per day) did not have a significantly higher CRC risk before or after adjustment for age, weight, height, smoking status, social class and intakes of energy, fibre, folate, red meat and processed meat (OR: 1.19; 95% CI: 0.75, 1.91). None of the other categories showed a significant association with CRC risk compared with the group consuming >0–<5 g per day of intake. No significant sex-specific associations were observed between alcohol intake and CRC risk. When we conducted further analyses adjusting for non-alcohol energy and the same covariates used in multivariable models, the results scarcely differed and they did not vary by sex.

In the sensitivity analysis in which further adjustment for physical activity and educational level was made in a subset of the study population with complete covariate information, being a non-drinker was significantly associated with an increased CRC risk. However, this result was seen under both multivariable models 1 and 2 in the sensitivity analysis, indicating that the result is not due to adjustment for physical activity and educational level but rather to the omission of some cohorts from the analysis. The point estimates for the highest category of alcohol intake tended to be higher in this subset of studies (OR: 1.41; 95% CI: 0.85, 2.34 for ⩾45 g per day). As in the main analyses, adjustment for physical activity and educational level did not alter the results in the subset.

Multivariable models 1 and 2 were suggestive of a J-shaped association between alcohol intake and CRC risk. However, a further analysis using continuous alcohol intake with a quadratic term provided no evidence for a non-linear association between alcohol intake and CRC risk (*P* for quadratic term=0.17 for drinkers). Additional adjustment for family history of CRC (343 cases and 1370 controls) did not make substantial differences to ORs (data not shown).

When we investigated these associations further by tumour subsites (stratified by sex), no clear associations were observed between risks of overall colorectum, proximal/distal colon, or rectum and alcohol intake in both sexes (Table 4). The analysis using multivariable model 2 for the subset of individuals with information on physical activity and educational level showed increased distal colon cancer risk for alcohol intake of ⩾30 g per day compared with intake of >0–<5 g per day (OR: 2.36; 95% CI: 1.13, 4.91, *P* trend for drinkers =0.03). However, this may be a chance finding.

Using data obtained from food diaries, we were also able to examine the association between specific alcoholic beverage intake and risk of CRC. When we calculated multivariable ORs per 1 s.d. increase in intake of beer (280 g), wine (70 g), spirits (20 g) and fortified wine (24 g), no clear associations were observed. The results did not vary by sex (data not shown).

**Figure 1:**
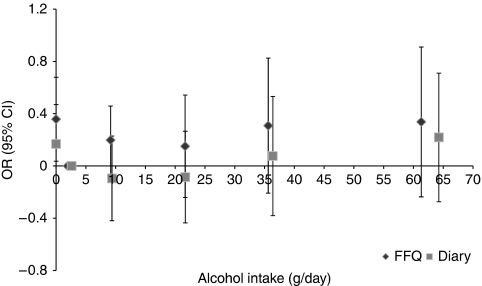
Comparison of odds ratios (ORs) in a log scale for categories for alcohol intake data (0, >0–<5 (reference), 5–<15, 15–<30, 30–<45 and ⩾45 g per day) obtained by food diaries or FFQs. A total of 2305 study participants had complete alcohol intake information from both diaries and FFQs (*n*=496 cases and 1809 controls). Ors for each category were plotted against the mean alcohol intake (g per day) for each category (0, 2.6, 9.4, 21.7, 36.4 and 64 g per day for food diaries and 0, 1.9, 9.1, 21.7, 35.6 and 61.3 g per day for FFQs, respectively) and were adjusted for age, weight, height, smoking status, social class, intakes of fibre and folate.

Table 5 and Figure 1 show a comparison of the results from using FFQ and food diary to obtain measurements of alcohol intake. Analyses using FFQ resulted in a similar pattern of associations to those using food diaries. The association between alcohol intake and CRC risk remains statistically non-significant using FFQ, although suggests an increasing trend in the OR estimates with increasing alcohol intake (*P* for trend=0.09 among drinkers in multivariable model 1). The distribution of participants across the categories of alcohol intake differed in the FFQ and food diary data. Among the subset of participants with both measurements (*n*=2305), out of 646 individuals who reported zero intake on the food diary, 305 (47%) reported being non-drinkers on the FFQ. Approximately 95% of individuals (*n*=613) reporting zero alcohol intake on the food diary consumed <5 g per day of alcohol according to the FFQ. A total of 67 individuals (18 %) reported zero alcohol intake on FFQ and >0 alcohol intake on the food diary.

Sex-specific analyses of the linear association between CRC risk and an increase in alcohol intake of 8 g per day (1 unit per day) showed no clear linear associations in either sex (OR: 0.99; 95% CI: 0.93, 1.05 for men, OR: 1.01; 95% CI: 0.92, 1.12 for women in multivariable model 1). The results scarcely differed from the analyses using drinkers only. When we examined interactions between alcohol intake and BMI (<25 kg m^−2^, ⩾25 kg m^−2^), smoking status (never, former or current), the *P*-values for the interaction were 0.26 for BMI and 0.53 for smoking status. The Reference Nutrient Intake in the United Kingdom for folate is 200 *μ*g per day (Department of Health, 1999). When folate intake was dichotomised below and above this level, the *P*-value for interaction was 0.59. There was no evidence of heterogeneity between centres in the association between alcohol intake and CRC risk in the different centres (*P*=0.30). Centre-specific ORs for CRC per 8 g per day of alcohol intake (1 unit per day) were computed (Supplementary Data). The summary OR estimate for 8 g per day increase in alcohol intake was derived by fixed effects meta-analysis and found to be 1.00 (95% CI: 0.95, 1.05) after adjusting for age, and intakes of energy, folate, fibre and red and processed meat.

## Discussion

In this large nested case–control study of 579 CRC cases and 1996 matched controls, alcohol intake within the observed range was not associated with a significantly increased CRC risk after multivariable adjustment when compared with alcohol intake of >0–<5 g per day. In subgroup analyses of cancer sites including proximal/distal colon and rectum, no clear associations were observed with total alcohol intake. There was also no evidence of a difference between men and women in the association between alcohol intake and CRC risk. Analyses using a subset of participants who had completed both FFQs and food diaries showed similar shaped associations using each of the two instruments, although risk estimates were higher but still statistically non-significant when using FFQ data.

A meta-analysis of prospective cohort studies showed 19% of increased risk of CRC with an increase of 100 g per week in alcohol intake (Moskal *et al*, 2006). Recent cohort studies in which FFQs were the main nutritional instrument have shown no association (Chen *et al*, 2005), or a significant adverse effect of alcohol when intake is greater than ∼16 g per day (Toriola *et al*, 2008), 30 g per day (Ferrari *et al*, 2007; Bongaerts *et al*, 2008; Mizoue *et al*, 2008) or ∼45 g per day (Akhter *et al*, 2007) compared with study-specific reference groups of lower intakes. These individual studies have not found consistent results in sex- and subsite-specific analyses, with several studies finding greater risk of rectal than colon cancer for alcohol intake of ⩾30 g per day (Ferrari *et al*, 2007; Bongaerts *et al*, 2008). The Million Women Study recently reported a positive association between moderate alcohol intake (>15 drinks per week) and rectal cancer risk but found no evidence of increased colon cancer risk among middle-aged women (Allen *et al*, 2009). Previous studies have, however, failed to reach clear consensus on the association between moderate alcohol drinking (<30 g per day) and colon or rectal cancer risk, and there are still few studies which have investigated proximal and distal colon cancer separately.

It has been suggested that the aetiology of CRC varies by subsite (Stang and Kluttig, 2008; Li and Lai, 2009). The proximal and distal colons have different embryonic origins and their physiology and functions may vary (Stang and Kluttig, 2008). Studies have also shown that microsatellite instability is often linked to proximal colon cancer, whereas chromosomal instability is more common in distal colon cancer (Lindblom, 2001). Therefore, subsite-specific studies are required for a better understanding of the aetiology of CRC. Our study, exploring CRC subsites in men and women in detail, suggested elevated risk of distal colon cancer, for individuals with alcohol intake of ⩾30 g per day compared with >0–<5 g per day and a possible dose–response relationship among drinkers when analysed for the subset of cohorts with complete covariate information. Thus, future studies are warranted focusing on a possible role of alcohol use in the risk of colon cancer, especially proximal or distal colon cancer.

The Panel of the WCRF/AICR Report judged that the evidence of alcohol intake of >30 g per day as a cause of CRC is convincing in men and probable in women (WCRF/AICR, 2007), based on a sex-specific meta-analysis finding summary effect estimates of 1.09 (95% CI: 1.02, 1.15) per 10 g per day increase in alcohol intake for men, based on 7 cohort studies, and 1.00 (95% CI: 0.89, 1.40) for women, based on 3 cohort studies. There were no statistically significant differences in association by cancer site. The threshold of 30 g per day of alcohol intake is from the results of the pooled analysis of eight cohort studies in which no increased risk was observed below the threshold (Cho *et al*, 2004).

Our results are consistent with the 2007 WCRF/AICR Report. We found no increased risk of CRC up to 30 g per day of alcohol intake, with no substantial differences detected in subsite-specific analyses. Although men and women have been shown to have different physiological responses to alcohol (Ely *et al*, 1999) and the effect of alcohol in our study seemed larger in men (OR: 1.24, 95% CI: 0.76–2.03 for drinkers with ⩾30 g per day compared with the lightest category drinkers (>0–<5 g per day)) than in women (OR: 1.03, 95% CI: 0.54–1.96 for drinkers with ⩾30 g per day compared with the lightest category of drinkers (>0–<5 g per day)), the associations were not statistically significant. We did not find differential associations with CRC risk by type of alcoholic beverage. This is consistent with the Report which judged that the causal factor is evidently alcohol itself, irrespective of the type of alcoholic drink. A limited number of studies were included in the meta-analysis of alcohol intake and CRC risk in the WCRF/AICR Report. Therefore, our findings contribute to update the current evidence for a future review, confirming no significantly increased risk of CRC with <30 g per day of alcohol intake.

The mechanism by which alcohol may influence CRC risk is not well understood (Stewart and Kleihues, 2003). Hypotheses include a local solvent action which facilitates absorption of other carcinogens, for example, a synergetic effect with tobacco smoking (Boffetta and Hashibe, 2006), and an indirect effect through associated deficiencies in nutrients, especially through changes in folate metabolism (Giovannucci *et al*, 1995). However, in our study, no significant interactions were observed between alcohol intake and folate intake or tobacco smoking with regard to CRC risk.

Our study has several strengths. Its prospective study design precluded bias attributable to differential recall of intake of alcohol by case status. We were able to examine the influence of alcohol intake on site- and sex-specific CRC risk. Furthermore, different types of alcoholic beverages from food diaries were assessed in association with CRC risk.

This study provided the measure of alcohol intake by using both food diaries and FFQs, whereas previous studies on alcohol and CRC risk have relied only on FFQs. The use of food diaries and FFQs for habitually consumed food items have been discussed (Bingham *et al*, 1997, 2003, 2008). However, there have been few direct attempts to compare those two different nutritional instruments prospectively for episodically consumed food items, including alcohol. Previous studies have shown that FFQs were not inferior in measuring alcohol intake relative to prospective food diaries (Feunekes *et al*, 1999), and FFQs showed a high level of reproducibility and validity compared with diet records as a reference method (Ferraroni *et al*, 1996). Our study, which has information both from food diaries and FFQs, found that although FFQs and food diaries cover different durations and measurements may differ between the two instruments, using well-constructed food diaries for measurement of infrequently consumed food items can provide results that do not differ substantially from those using FFQs.

This study used original data from seven UK mature cohorts with standardised diary data entry, which enabled us to create identical categories for alcohol intake across studies that were in line with previous studies (Cho *et al*, 2004), removing some potential sources of heterogeneity across studies. Furthermore, we were able to adjust for a range of known confounding factors.

An important limitation of this study is that we were unable to differentiate life-long abstainers and former drinkers in the category of non-drinkers in either FFQs or diaries. As previously discussed, many non-drinkers may be former drinkers who had given up drinking because of incipient disease (Doll *et al*, 1994), although a sensitivity analysis excluding a further 111 cases incident within 3 years of diary completion did not materially change our results. Moreover, in the 4–7-day diaries, we were unable to differentiate non-drinkers from episodic drinkers who happened not to consume alcohol during the time period covered by the diary. Hence, it is likely that the ‘non-drinker’ category in our diary analyses contains participants who were actually drinkers at the time when diaries were administered. In light of this, we focused on analyses from non-zero alcohol drinkers and reported trend tests for drinkers separately (Allen *et al*, 2009). We found a moderate positive but non-significant CRC risk in those consuming ⩾30 g per day of alcohol using data from both food diaries and FFQs. However, in our study, almost half of the participants reported drinking <5 g per day in both food diaries and FFQs and only 19% of men and 17% of women reported intake in excess of the recommended daily maxima of 3–4 units (<32 g) daily for men and 2–3 units (<24 g) daily for women. Insufficient participants in the heavier categories prevented us from estimating any potential effect of high alcohol intake with sufficient precision.

Another limitation was that alcohol intake was assessed only once by self-report. As heavy alcohol drinking is considered to be unhealthy, it is likely that individuals underreport their alcohol intake, particularly in the case of heavy intake (Rehm *et al*, 1999), resulting in overestimation of the actual carcinogenic effect of the habit. In addition, drinking habits are liable to change throughout the lifetime. However, we conducted a sensitivity analysis using data from the EPIC-Norfolk cohort in which information on alcohol intake from participants recalling their habits at ages 20 and 30 years is available, and we again did not find any evidence of an association with CRC risk, although participants tended to report higher alcohol intake at younger ages (data not shown). Nonetheless, more research with additional information on alcohol intake over a longer period of time and on specific drinking behaviour such as binge drinking is required to clarify any hazardous effect of excessive alcohol drinking on CRC risk.

In summary, we found no increased risk of CRC up to 30 g per day of alcohol intake within the UK Dietary Cohort Consortium. However, because of an insufficient number of participants in the heavier categories, a modest increased risk in those consuming ⩾30 g per day cannot be excluded. Excessive alcohol intake has been causally related to numerous medical conditions (Rehm *et al*, 2003). Drinking-related morbidity and mortality constitute a large burden of diseases in Europe and worldwide (Ezzati *et al*, 2004; Rehm *et al*, 2006). In the United Kingdom, there was a substantial increase in both hospital admissions and deaths specifically related to alcohol misuse between 1991 and 2007, costing over £2.7 billion to the National Health Service annually (Rachel *et al*, 2009). The risks of alcohol intake should therefore be carefully considered in any decisions about alcohol drinking.

## Figures and Tables

**Table 1:** Description of studies participating in the UK Dietary Cohort Consortium and summary of alcohol intake among colorectal cancer cases and matched controls^a^

				**CRC cases**		**CRC controls**		**Mean alcohol intake for cases (s.d.)**		**Mean alcohol intake for controls (s.d.)**	
**Study**	**Age range at baseline (years)**	**Size of the cohort at baseline**	**Assessment of alcohol intake**	**Men**	**Women**	**Men**	**Women**	**Men**	**Women**	**Men**	**Women**
EPIC-Norfolk	40–77	25 000	7DD/FFQ	179	139	716	556	15.2 (18.6)	6.7 (11.9)	15.5 (21.1)	7.2 (10.8)
EPIC-Oxford	32–84	65 429	7DD/FFQ	39	82	87	193	17.0 (17.5)	8.4 (11.3)	21.0 (27.8)	8.1 (10.8)
Guernsey Study	39–78	6127	4DD	N/A	28	N/A	55	N/A	7.6 (10.4)	N/A	6.0 (10.8)
Oxford Vegetarian Study	26–79	11 140	4DD	7	24	16	54	6.3 (7.3)	7.7 (15.9)	9.8 (12.5)	9.1 (13.3)
MRC National Survey of Health and Development (NSHD)	43	5362	7DD	4	3	16	12	39.1 (66.1)	16.4 (14.3)	28.9 (23.7)	13.6 (11.6)
UK Women's Cohort Study (UKWCS)	44–78	35 792	4DD/FFQ	N/A	25	N/A	100	N/A	9.3 (13.9)	N/A	8.1 (11.3)
Whitehall II	41–62	10 308	7DD/FFQ	37	12	145	46	25.8 (26.3)	18.2 (17.2)	22.2 (20.4)	8.8 (10.4)

Abbreviations: CRC=colorectal cancer; EPIC=European Prospective Investigation into Cancer and Nutrition; MRC=Medical Research Council; 7DD=7-day food diary; 4DD=4-day food diary; FFQ=food frequency questionnaire; N/A=not applicable.

a A *t*-test indicated that there was no statistically significant difference in mean alcohol intake between cases and controls.

**Table 2:** Distribution of participant characteristics by categories of alcohol intake as assessed by food diaries, shown separately for men and women^a^

	**Baseline alcohol intake**						
	**Non-drinkers**	**>0–<5 g per day**	**5–<15 g per day**	**15–<30 g per day**	**30–<45 g per day**	**⩾45 g per day**	***P*-value** b
*All*							
Cases/controls (*n*)	187/574	112/405	116/443	86/328	40/135	38/111	
							
*Men*							
Cases/controls (*n*)	68/200	39/175	55/224	45/188	28/96	31/97	
Alcohol at baseline (g per day)	0.0	2.7 (1.4)	9.5 (2.9)	21.8 (4.2)	36.6 (4.2)	66.1 (24.4)	<0.001
Age (years)	64.2 (8.4)	63.1 (8.3)	61.7 (9.8)	61.5 (9.1)	60.0 (8.5)	59.9 (9.2)	<0.001
Height (m)	1.73 (0.1)	1.73 (0.1)	1.75 (0.1)	1.75 (0.1)	1.75 (0.1)	1.75 (0.1)	<0.001
Weight (kg)	77.9 (11.7)	80.8 (13.2)	78.1 (11.2)	79.7 (10.3)	80.7 (11.1)	82.4 (11.6)	<0.001
BMI (kg m^−2^)	26.1 (3.4)	26.9 (4.0)	25.6 (3.2)	26.1 (3.0)	26.2 (2.9)	26.8 (3.0)	<0.001
							
*Cigarette smoking status* (%)^c^							
Never	41.1	32.7	35.4	35.2	33.1	21.4	0.001
Former	49.1	60.7	57.4	55.4	53.2	60.3	
Current	9.8	6.6	7.2	9.4	13.7	18.3	
Total energy (kcal)^d^	2077 (546)	2117 (470)	2218 (479)	2225 (486)	2345 (478)	2478 (545)	<0.001
							
*Physical activity* (%)^c^,e							
Low	67.3	56.9	60.9	47.6	61.7	63.1	0.001
High	32.7	43.1	39.1	52.4	38.3	36.9	
							
*Educational level* (%)^c^,f							
Low	58.0	43.1	41.3	42.3	37.9	33.9	<0.001
High	42.0	56.9	58.7	57.7	62.1	66.1	
Social class^c^,g							
Non-manual	50.2	59.6	64.3	76.3	80.0	77.0	<0.001
Manual	49.8	40.4	35.7	23.7	20.0	23.0	
							
*Family history of colorectal cancer* (%)^c^							
No	93.5	91.1	94.2	95.5	91.9	93.6	0.69
Yes	6.5	8.9	5.8	4.5	8.1	6.4	
Folate intake (*μ*g per day)	282 (90)	274 (69)	293 (82)	294 (85)	308 (86)	314 (92)	<0.001
Fibre intake (g per day)	17 (7)	17 (6)	17 (6)	16 (6)	16 (5)	14 (6)	<0.001
Red meat intake (g per day)	33 (30)	34 (25)	39 (30)	40 (27)	40 (30)	46 (34)	<0.001
Processed meat intake (g per day)	24 (24)	27 (22)	25 (20)	29 (24)	27 (23)	30 (22)	0.02
							
*Women*							
Cases/controls (*n*)	119/374	73/230	61/219	41/140	12/39	7/14	
Alcohol at baseline (g per day)	0.0 (0.0)	2.5 (1.4)	9.4 (2.9)	21.5 (4.5)	35.3 (3.8)	56.9 (11.3)	<0.001
Age (years)	63.1 (9.7)	62.5 (9.0)	60.2 (9.5)	58.7 (10.6)	57.8 (10.9)	59.3 (11.5)	<0.001
Height (m)	1.60 (0.07)	1.61 (0.06)	1.62 (0.07)	1.62 (0.06)	1.64 (0.06)	1.60 (0.05)	<0.001
Weight (kg)	66.8 (13.1)	66.9 (11.7)	66.5 (11.0)	65.4 (10.9)	66.7 (10.1)	61.2 (9.4)	0.27
BMI (kg m^−2^)	26.1 (4.8)	25.9 (4.3)	25.5 (4.0)	24.9 (4.1)	24.8 (3.3)	23.9 (3.7)	0.005
							
*Cigarette smoking status* (%)^c^							
Never	64.5	59.5	57.1	53.7	40.0	19.1	<0.001
Former	27.8	33.1	33.2	37.3	38.0	61.9	
Current	7.6	7.4	9.6	9.0	22.0	19.1	
Total energy (kcal)^d^	1639 (418)	1653 (334)	1747 (369)	1803 (372)	1909 (354)	1916 (269)	<0.001
							
*Physical activity* (%)^c^,e							
Low	73.7	68.1	66.0	68.6	73.8	65.0	0.33
High	26.4	32.0	34.0	31.5	26.2	35.0	
							
*Educational level* (%)^c^,f							
Low	71.6	69.0	58.2	60.9	47.9	27.8	<0.001
High	28.4	31.0	41.8	39.1	52.1	72.2	
Social class^c^,g							
Non-manual	69.0	72.1	79.6	85.6	83.3	95.2	<0.001
Manual	31.0	27.9	20.4	14.4	16.7	4.8	
							
*Family history of colorectal cancer* (%)^c^							
No	91.4	93.9	87.4	93.9	93.3	100.0	0.20
Yes	8.6	6.1	12.7	6.1	6.7	0.0	
Folate intake (*ì*g per day)	246 (78)	247 (70)	252 (70)	250 (73)	251 (65)	241 (72)	0.93
Fibre intake (g per day)	15 (6)	15 (5)	15 (5)	15 (5)	14 (4)	12 (5)	0.14
Red meat intake (g per day)	25 (26)	28 2(6)	29 (26)	33 (32)	41 (36)	38 (25)	0.002
Processed meat intake (g per day)	16 (18)	15 (15)	15 (15)	17 (17)	17 (16)	16 (19)	0.96

Abbreviation: BMI=body mass index.

a Mean (s.d.) or number (%), and *P*-values for tests of association.

b For continuous variables, analysis of variance or a Kruskal–Wallis test (for red meat and processed meat intake) was used to test whether the variables differed significantly across categories of alcohol intake. For categorical variables, *χ*^2^ tests were used to assess association with alcohol intake.

c Numbers do not sum to the total number of participants due to missing data.

d Total energy includes energy from alcohol.

e Low physical activity was defined as being inactive or moderately inactive, and high physical activity was defined as being moderately active or active.

f Educational levels were regrouped into low educational level (no qualification or General Certificate of Secondary Education (GCSE) level or equivalent) and high educational level (degree or equivalent, A-level or equivalent).

g Social class was classified according to the Registrar General's occupation-based classification scheme and was dichotomised into non-manual (social class I, II and IIInm) and manual (IIIm, IV and V).

**Table 3:** Odds ratios (95% confidence intervals) from multivariable models for colorectal cancer risk in categories of total alcohol intake as assessed by food diaries

	**Alcohol intake (g per day)**							
	**Non-drinkers**	**>0–<5 g per day**	**5–<15 g per day**	**15–<30 g per day**	**30–<45 g per day**	**⩾45 g per day**	***P* for trend**	***P* trend for drinkers**
*Main models* a								
No. of all participants	761	517	559	414	175	149		
Colorectal cancer cases	187	112	116	86	40	38		
Age-adjusted model^b^	1.15 (0.88–1.51)	1.00	0.93 (0.69–1.26)	0.93 (0.68–1.28)	1.13 (0.74–1.72)	1.29 (0.83–2.01)	0.79	0.31
Multivariable model 1^c^	1.16 (0.88–1.53)	1.00	0.91 (0.67–1.24)	0.90 (0.65–1.25)	1.02 (0.66–1.58)	1.19 (0.75–1.91)	0.82	0.44
Male	1.53 (0.98–2.41)	1.00	1.06 (0.66–1.69)	1.02 (0.63–1.66)	1.20 (0.68–2.12)	1.24 (0.69–2.22)	0.97	0.21
Female	1.00 (0.70–1.42)	1.00	0.84 (0.56–1.26)	0.87 (0.55–1.37)	0.90 (0.43–1.87)	1.52 (0.56–4.10)	0.72	0.97
								
*Sensitivity analysis* d								
Multivariable model 1^c^	1.48 (1.08–2.03)	1.00	0.94 (0.66–1.33)	1.00 (0.69–1.45)	1.21 (0.75–1.96)	1.41 (0.85–2.34)	0.79	0.22
Multivariable model 2^e^	1.49 (1.08–2.05)	1.00	0.93 (0.65–1.33)	0.98 (0.68–1.43)	1.23 (0.76–1.99)	1.39 (0.83–2.32)	0.82	0.17

a Main conditional logistic regression models: all participants (579 cases and 1996 controls); *P*-values for trend were drawn from tests for trend by modelling alcohol intake as a continuous variable in a conditional logistic regression analysis, whereas *P*-values for trend for drinkers were drawn from tests for trend only from non-zero alcohol drinkers.

b Age adjusted.

c Adjusted for age, weight, height, smoking status, social class, intakes of energy, fibre, folate, red meat and processed meat.

d Sensitivity analyses: restricted to individuals with complete covariates information (458 cases and 1734 controls).

e Adjusted for age, weight, height, physical activity, educational level, smoking status, social class, intakes of energy, fibre, folate, red meat and processed meat.

**Table 4:** Odds ratios (95% confidence intervals) by subsite of colorectal cancer according to alcohol intake

	**Overall colorectum**				**Colon**				**Proximal colon**				**Distal colon**				**Rectum**			
	**Cases**	**OR (95% CI)**	***P* for trend**	***P* trend for drinkers**	**Cases**	**OR (95% CI)**	***P* for trend**	***P* trend for drinkers**	**Cases**	**OR (95% CI)**	***P* for trend**	***P* trend for drinkers**	**Cases**	**OR (95% CI)**	***P* for trend**	***P* trend for drinkers**	**Cases**	**OR (95% CI)**	***P* for trend**	***P* trend for drinkers**
*Multivariable model* a																				
*All participants*																				
Non-drinkers	187	1.16 (0.88–1.53)	0.72	0.69	122	1.18 (0.83–1.66)	0.85	0.63	60	1.26 (0.81–1.97)	0.54	0.59	46	0.97 (0.60–1.56)	0.46	0.17	65	1.10 (0.68–1.78)	0.76	0.86
>0–<5 g per day	112	1.00 (Reference)			74	1.00 (Reference)			33	1.00 (Reference)			29	1.00 (Reference)			38	1.00 (Reference)		
5–<30 g per day	202	0.91 (0.69–1.19)			132	0.88 (0.63–1.22)			58	0.88 (0.57–1.34)			48	0.91 (0.58–1.45)			70	0.97 (0.60–1.58)		
⩾30 g per day	78	1.09 (0.76–1.58)			52	1.21 (0.77–1.90)			23	1.03 (0.57–1.86)			23	1.60 (0.85–3.01)			26	0.93 (0.48–1.78)		
																				
*Male participants*																				
Non-drinkers	68	1.52 (0.97–2.39)	0.90	0.23	46	1.82 (1.02–3.22)	0.69	0.21	23	2.46 (1.10–5.51)	0.40	0.28	20	1.22 (0.56–2.65)	0.86	0.42	22	1.14 (0.53–2.43)	0.93	0.90
>0–<5 g per day	39	1.00 (Reference)			26	1.00 (Reference)			20	1.00 (Reference)			19	1.00 (Reference)			13	1.00 (Reference)		
5–<30 g per day	100	1.04 (0.68–1.58)			61	1.05 (0.62–1.77)			28	1.41 (0.67–2.97)			22	0.83 (0.42–1.66)			39	1.10 (0.53–2.26)		
⩾30 g per day	59	1.24 (0.76–2.03)			39	1.49 (0.81–2.74)			20	1.93 (0.83–4.50)			16	1.16 (0.50–2.66)			20	1.04 (0.43–2.51)		
																				
*Female participants*																				
Non-drinkers	119	1.00 (0.70–1.43)	0.54	0.63	76	0.93 (0.60–1.46)	0.55	0.73	37	0.93 (0.53–1.63)	0.16	0.14	26	0.88 (0.46–1.67)	0.19	0.27	43	1.15 (0.60–2.19)	0.84	0.97
>0–<5 g per day	73	1.00 (Reference)			48	1.00 (Reference)			23	1.00 (Reference)			16	1.00 (Reference)			25	1.00 (Reference)		
5–<30 g per day	102	0.85 (0.59–1.23)			71	0.81 (0.51–1.27)			30	0.73 (0.42–1.28)			26	1.05 (0.55–2.00)			31	0.94 (0.48–1.86)		
⩾30 g per day	19	1.03 (0.54–1.96)			13	1.09 (0.49–2.42)			3	0.52 (0.17–1.61)			7	3.34 (1.11–10.02)			6	0.95 (0.30–2.95)		
																				
*Multivariable model* b																				
*All participants*																				
Non-drinkers	147	1.49 (1.08–2.05)	0.93	0.32	99	1.63 (1.09–2.43)	0.67	0.20	57	1.99 (1.18–3.34)	0.76	0.77	34	1.31 (0.73–2.34)	0.08	0.03	48	1.23 (0.70–2.17)	0.67	0.73
>0–<5 g per day	84	1.00 (Reference)			55	1.00 (Reference)			28	1.00 (Reference)			23	1.00 (Reference)			29	1.00 (Reference)		
5–<30 g per day	156	0.95 (0.70–1.30)			107	1.00 (0.68–1.47)			49	0.97 (0.59–1.61)			39	1.23 (0.71–2.12)			49	0.86 (0.49–1.53)		
⩾30 g per day	71	1.30 (0.86–1.95)			47	1.47 (0.89–2.43)			20	1.25 (0.63–2.47)			23	2.36 (1.13–4.91)			24	1.01 (0.48–2.11)		
																				
*Male participants*																				
Non-drinkers	61	1.64 (1.01–2.66)	0.76	0.16	42	2.08 (1.14–3.82)	0.53	0.14	23	2.80 (1.17–6.67)	0.57	0.41	17	1.44 (0.63–3.29)	0.63	0.21	19	1.04 (0.43–2.51)	0.96	0.73
>0–<5 g per day	34	1.00 (Reference)			23	1.00 (Reference)			9	1.00 (Reference)			12	1.00 (Reference)			11	1.00 (Reference)		
5–<30 g per day	90	1.05 (0.67–1.64)			58	1.13 (0.65–1.95)			27	1.43 (0.64–3.18)			20	0.98 (0.47–2.06)			32	0.99 (0.43–2.28)		
⩾30 g per day	56	1.36 (0.80–2.30)			37	1.65 (0.86–3.14)			19	1.78 (0.71–4.46)			16	1.64 (0.64–4.16)			19	1.06 (0.39–2.92)		
																				
* Female participants*																				
Non-drinkers	86	1.34 (0.87–2.08)	0.59	0.82	57	1.31 (0.76–2.28)	0.75	0.92	34	1.65 (0.81–3.35)	0.19	0.10	17	1.05 (0.43–2.57)	0.09	0.26	29	1.44 (0.64–3.25)	0.77	0.83
>0–<5 g per day	50	1.00 (Reference)			32	1.00 (Reference)			19	1.00 (Reference)			11	1.00 (Reference)			18	1.00 (Reference)		
5–<30 g per day	66	0.82 (0.52–1.30)			49	0.88 (0.50–1.54)			22	0.71 (0.34–1.49)			19	1.56 (0.63–3.84)			17	0.65 (0.27–1.59)		
⩾30 g per day	15	1.19 (0.56–2.53)			10	1.20 (0.46–3.13)			1	0.50 (0.11–2.37)			7	3.76 (0.92–15.37)			5	1.53 (0.40–5.91)		

Abbreviations: OR=odds ratio; CI=confidence interval.

a Age, weight, height, smoking status, social class, intakes of energy, fibre, folate, red meat and processed meat, adjusted (579 cases and 1996 controls).

b Age, weight, height, physical activity, educational level, smoking status, social class, intakes of energy, fibre, folate, red meat and processed meat, adjusted (458 cases and 1734 controls).

**Table 5:** Odds ratios (95% confidence intervals) from multivariable models for colorectal cancer risk in categories of alcohol intake as assessed by food diaries and FFQs among participants with both measures^a^

	**Alcohol intake (g per day)**							
	**Non-drinkers**	**>0–<5 g per day**	**5–<15 g per day**	**15–<30 g per day**	**30–<45 g per day**	**⩾45 g per day**	***P* for trend**	***P* trend for drinkers**
*Food diaries*								
No. of all participants	646	477	510	371	165	136		
Colorectal cancer cases	149	100	100	75	38	34		
Multivariable model 1^b^	1.18 (0.88–1.60)	1.00	0.91 (0.66–1.26)	0.92 (0.65–1.30)	1.08 (0.68–1.70)	1.24 (0.76–2.04)	0.97	0.60
Multivariable model 2^c^	1.38 (1.00–1.91)	1.00	0.90 (0.63–1.29)	0.98 (0.67–1.42)	1.20 (0.74–1.95)	1.32 (0.79–2.22)	0.84	0.25
								
*FFQs*								
No. of all participants	372	867	662	226	100	78		
Colorectal cancer cases	84	171	150	46	26	19		
Multivariable model 1^b^	1.43 (1.04–1.97)	1.00	1.22 (0.94–1.58)	1.16 (0.79–1.72)	1.36 (0.81–2.28)	1.40 (0.79–2.49)	0.12	0.09
Multivariable model 2^c^	1.33 (0.96–1.86)	1.00	1.16 (0.87–1.53)	1.07 (0.71–1.61)	1.18 (0.68–2.03)	1.30 (0.72–2.38)	0.36	0.07

Abbreviation: FFQ=food frequency questionnaire.

a Conditional logistic regression analyses were restricted to participants who completed both the FFQ and the food diary (496 cases and 1809 controls). Owing to missing information in FFQ data, models were not adjusted for intakes of energy, red meat and processed meat. Adjusting for these variables in models using diary information did not alter the results. *P*-values for trend were drawn from tests for trend by modelling alcohol intake as a continuous variable in a conditional logistic regression analysis, whereas *P*-values for trend for drinkers were drawn from tests for trend only from non-zero alcohol drinkers.

b Adjusted for age, weight, height, smoking status, social class, intakes of fibre and folate adjusted in the main model (496 cases and 1809 controls).

c Adjusted for age, weight, height, physical activity, educational level, smoking status, social class, intakes of fibre and folate in the sensitivity analyses restricted to individuals with complete covariate information (442 cases and 1701 controls).
