# Supplementary material for: Alcohol intake and risk of colorectal cancer: Results from the UK Dietary Cohort Consortium
Source: Br J Cancer. 2010 Jul 20;103(5):747–56. doi: 10.1038/sj.bjc.6605802 (PMC2938250; doi:10.1038/sj.bjc.6605802)
Supplement: Supplementary Data and Appendix 1 [file 6605802x1.doc]

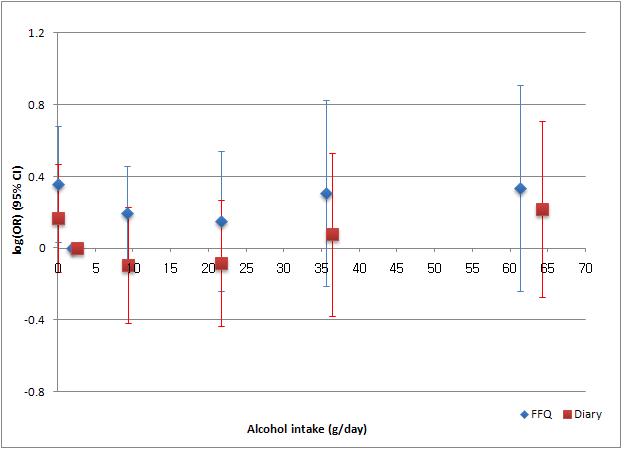


**Appendix 1** Comparison of odds ratios in a log scale for categories for alcohol intake data (0, >0 to <5 (reference), 5 to <15, 15 to <30, 30 to <45, and ≥45 g/day) obtained by food diaries or by FFQ. A total of 2,305 study participants had complete alcohol intake information from both diaries and FFQ (n=496 cases, 1,809 controls). Odds ratios for each category were plotted against the mean alcohol intake (g/day) for each category (0, 2.6, 9.4, 21.7, 36.4, and 64 g/day for food diaries and 0, 1.9, 9.1, 21.7, 35.6, and 61.3 g/day for FFQ, respectively) and were adjusted for age, weight, height, smoking status, social class, intakes of fibre, and folate.

**Supplementary data** Centre specific odds ratios (95% confidence intervals) for colorectal cancer per 8 g/day (1 unit/day) of alcohol intake. All participants (579 cases, 1,996 controls) were included and odds ratios were adjusted for age, intakes of energy, folate, fibre, and red and processed meat. The summary estimate was derived by fixed effects meta-analysis.
